# A Structural Analysis of Gender-Based Violence and Depression in the Lives of Sexual Minority Women and Trans People

**DOI:** 10.1177/08861099231155887

**Published:** 2023-02-28

**Authors:** Charmaine C. Williams, Margaret F. Gibson, Emily Mooney, Joellean R. Forbes, Deone Curling, datejie cheko green, Lori E. Ross

**Affiliations:** 1 Factor-Inwentash Faculty of Social Work, 7938 University of Toronto, Toronto, Ontario, Canada; 2 School of Social Work, 141634 Renison University College, University of Waterloo, Waterloo, Ontario, Canada; 3 Allied Psychological Services, Toronto, Canada; 4 Independent Scholar, Toronto, Canada; 5 Dalla Lana School of Public Health, 7938 University of Toronto, Toronto, Ontario, Canada

**Keywords:** gender-based violence, gender expressions, mental health, qualitative, sexual orientation and LGBTQ issues

## Abstract

This article explores structural mechanisms that are the context for violence and depression in the lives of sexual minority women and trans people in Ontario, Canada. The article draws on interviews with 14 people who reported experiences of depression in the previous year, foregrounding three representative narratives. Narrative and case study analysis reveal that violence is a repeated and cumulative experience over lifetimes, occurring across different interpersonal contexts and institutional encounters. A common theme across the narratives is that experiences of violence are connected to a broader context in which structural arrangements, cultural norms, and institutional processes create conditions where marginalized people are put in harm's way, perpetrators are empowered, and justice and access to help are elusive. As the violence experienced by these sexual minority women and trans people is rooted in structural and cultural oppression represented in poverty, racism, misogyny, homophobia, and transphobia, the prevention of violence and its consequences for these and other marginalized populations requires systemic transformation of the structures and systems that currently allow and perpetuate harm.

Sexual minority women and trans people are known to be disproportionately victimized by gender-based violence (Rothman et al., 2011; Stotzer, 2009; Valentine et al., 2017). Further, there are well-established links between exposure to gender-based violence and mental health disorder for the same populations (Ali et al., 2022; Lombardi et al., 2002; Malta et al., 2020; Testa et al., 2012). Yet, the literature that establishes these associations does not address the social and cultural mechanisms that may explain them. The analysis for this article was undertaken to address the need to explicate the structural context for violence and associated depression in the lives of sexual minority women and trans people. Such analyses are needed to inform critical, empowering social work with victims of violence, and advocacy for transformations that will remove people from harm's way.

Young (1990) made the argument that violence is a component of the oppression of targeted groups, specifically:

What makes violence a face of oppression is less the particular acts themselves, though these are often utterly horrible, than the social context surrounding them, which makes them possible and even acceptable. What makes violence a phenomenon of social injustice, and not merely an individual moral wrong, is its systemic character, its existence as a social practice … it is a social given that everyone knows happens and will happen again (Young, 1990, pp. 61–62).

If violence is considered in social context, it is revealed as embedded in a structure represented by laws, policies, and institutional processes that create circumstances of disempowerment and risk for marginalized people (Decker et al., 2022; Montesanti & Thurston, 2015). This structural analysis aims to contribute to understandings of violence and depression as outcomes of such structural processes by revealing how social and institutional circumstances can converge to create the possibilities for sexual minority women and trans people to be targets of violence, and to experience further harm in the form of poor mental health. In doing so, it traces the path from structural violence to gender-based violence against lesbian, bisexual, and trans bodies, and long-term wounds of depression.

## Literature Review

Gender-based violence includes physical, sexual, psychological, emotional, or other types of harm or threats directed at an individual based on gender (Decker et al., 2022). Research examining violence in the lives of lesbian and bisexual women reveals that sexual minority women report high levels of lifetime exposure to sexual victimization, sexual harassment, and assault (Johnson & Grove, 2017; Rothman et al., 2011). Research has also shown that sexual minority women and racial minority women across sexual identities have more lifetime experiences of harassment and assault than women who are White, cisgender, and heterosexual (Katz-Wise & Hyde, 2012; Logie et al., 2014; SteelFisher et al., 2019). Similarly, trans people experience particularly high rates of victimization and can be victims of violence throughout their lives, targeted by a variety of perpetrators in multiple contexts (Bauer & Scheim, 2015; Stotzer, 2009; Testa et al., 2012; Valentine et al., 2017).

Like violence, depression is a common occurrence in the lives of oppressed people. The abundance of depression and despair among people who exist in conditions of social, economic, and political marginalization is testimony to the individual consequences of structural determinants (Burnette et al., 2019; Carr et al., 2014; Neitzke, 2016). Depression and violence are intertwined outcomes of structural conditions that jeopardize the well-being of marginalized people (DeVerteuil, 2015; Flynn et al., 2018). Yet, structural and cultural contexts allow violence against people in marginalized groups to be defined within individual contexts while muting structural contributions that implicate society as a whole (Gringeri & Roche, 2010; Montesanti & Thurston, 2015). Similarly, depression is defined as a disorder of psychology or neural pathways, diverting attention away from social and structural causes (Neitzke, 2016). Attending to the physical and mental health consequences of violence is vital work; however, it is equally vital to trace the path from wounded bodies to the institutions, cultural norms, and structures that are the origins of those wounds. Statistics of violence experienced by those who are victimized based on “gender role transgressions” (Russo & Pirlott, 2006) serve to identify the threat of violence faced by some, but accounts that explicate the structural dynamics implicated in that violence are needed to enact institutional practices that will prevent further harm. In the absence of such accounts, the scope for social work interventions is narrowed to support for those that have been victimized. Research that situates this violence in cultural and structural contexts builds the evidence for a broader scope of practice that includes advocacy, reform, civic and global engagement, and rebuilding of environments to promote safety and well-being for sexual and gender minority people.

One of the limitations of available knowledge about violence against sexual minority women and trans people is that it may obscure the experiences of people with intersecting identities. Although there are shared experiences within and across identity categories, the experiences that are associated with intersecting identities defined by race, (dis)ability, class, and other factors can confer specific risks to harm (Collins & Bilge, 2020; Crenshaw, 1991). For example, sexual and gender minority migrants report persistent experiences of violence before, during, and after immigration (Alessi et al., 2021) and racial and ethnic minority trans women describe needing strategies to withstand the pervasive threat of sexual violence (Hawkey et al., 2021). Intersectionality is an analytic tool that clarifies that the specific risks that are faced by people are influenced by how multiple identities correspond to layered experiences of discrimination and privilege (Few-Demo, 2014). Therefore, the overlooked experiences of racialized and other people in research about sexual minority women and trans people need to be surfaced to have an equitable understanding of violence faced by these populations. Furthermore, it is not sufficient to do research that associates violence with factors like race, sexual identity, and gender identity, because that maintains focus on individuals and veils the role of structural violence and associated social and institutional arrangements that cause harm (Farmer et al., 2006). The vulnerability is not the race, sexuality, or gender of the bodies but structural and systemic racism, sexism, cisheterosexism, etc., and the intersecting vectors of oppression in the environments these bodies must navigate. Structural violence and structural determinants create the possibility of violence before individuals are harmed (De Maio & Ansell, 2018).

Although this article focuses on the experiences of sexual minority women and trans people who have experienced violence and depression, its focus is not victimhood, or abusers or mental illness. Instead, this article focuses on survivors of violence who bear harm and injury that manifest as depression, and the systems, structural and cultural mechanisms that make misogynistic, homophobic, and transphobic violence against lesbian/bisexual women and trans people possible.

## Methods

### Study Design

The narratives in this article are drawn from the Pathways Project, a study of access to mental health treatment for lesbian, bisexual, and transgender people in Ontario, Canada (Ross et al., 2018; Steele et al., 2017; Williams et al., 2017). The research team and advisory board included university-based researchers, community-based service providers, and advocates who were also members of the communities that were the focus of the study. The project was approved by the research ethics boards for the University of Toronto and the Centre for Addiction and Mental Health in Toronto, Canada. The study included a survey (n = 704) and qualitative interviews (n = 24) with participants who completed the survey indicating experience of depression in the past 12 months. The survey asked questions about personal health circumstances, mental health supports, and life experiences, including experiences of discrimination. The interviews addressed the same issues in more detail, including questions about experiences of violence, as several survey respondents reported being victims of violence.

### Recruitment and Participants

Participants for the study were recruited through outreach to agencies, health services, community organizations, and online networks serving women and lesbian, gay, bisexual, transgender, or queer (LGBTQ) people. In keeping with the study's attention to intersectional experiences, the outreach for participants included engagement with agencies and networks serving a diverse range of racial and ethnic communities. Communications about the study emphasized the desire to understand how sexual and/or gender identities intersect with other identities, like race and class, and how they affect people's encounters with seeking and receiving mental health care (https://lgbtqhealth.ca/projects/pathways.php)*.* To be eligible for the study, participants had to be 18 years of age or older, living in Ontario, sufficiently fluent in English, and self-identifying as a woman and/or trans person.

Screening for eligibility to participate in the survey was accomplished within the online platform. Potential interview participants were selected from the pool of survey participants who indicated willingness to be contacted for an interview. A telephone screening interview confirmed eligibility, gathered information about sociodemographic characteristics (by asking people how they chose to identify within the categories), and included assessing the experience of depression in the past 12 months using the Composite International Diagnostic Interview (Wittchen, 1994). Heterosexual women participated in the survey but recruitment for the interview phase of the study was narrowed to focus on the experiences of lesbian, bisexual, and trans people representing Indigenous, racial minority, and White identities, and a range of socioeconomic statuses, geographic locations (urban, suburban, rural Ontario), and experiences with receiving mental health care. Interview participants could choose to receive a $10 gift card as an honorarium or direct the honorarium to a registered charitable organization.

The sample for this study was created by conducting a preliminary analysis of the twenty-four interviews based on Young's (1990) framework, then creating a subsample for for a secondary analysis (n = 14) by selecting transcripts of participants identified as making connections between violence and mental health. Most of the 14 participants self-identified as White (10 of 14), and all were adults between the ages of 25 and 55 years. Eight participants identified as women and six identified as trans people (including, but not limited to, identities as transgender, nonbinary, genderqueer, etc.). Eight participants were living in large urban centers and six described themselves as living in a suburban or rural environment.

### Data Analysis

The 14 interviews had been transcribed verbatim and were analyzed using a narrative analysis approach (Crabtree & Miller, 1992) and a code manual developed from the integration of theoretical concepts derived from Young's (1990) framework of the Five Faces of Oppression, Farmer et al.’s (2006) description of structural violence, and Crenshaw's (1991) articulation of intersectionality. The theoretical tenets of structural violence and intersectionality enhanced Young's conceptualization of violence, informing the interpretation of connections between experiences, cultural and structural contexts, and intersecting vectors of oppression. In addition, the first author used elements of case study method (Baxter & Jack, 2008), integrating within and across-case analysis to further explore participants’ descriptions of the context surrounding exposures to violence and its mental health consequences. As the analysis progressed, interpretations and examples were shared with research team members to develop consensus around emerging concepts. As the team included researchers, service providers, and service users/advocates representing the communities engaged through the research study, their perspectives and these discussions were important to establishing rigor, credibility, and reliability in the analysis.

This article foregrounds the narratives of three participants who exemplified broader themes revealed in the analysis across transcripts. This method was chosen to present a contextualized accounting of a few participant experiences while using across-case analysis to articulate the connections and divergences between their experiences and those of others positioned similarly. Pseudonyms are selected for the people described in the case studies and identifying characteristics of all people presented in the findings section are derived from the language people chose to describe themselves.

## Findings

Although most of the people represented in this analysis reported White-identified racial identities, the integration of an intersectionality analysis led to foregrounding narratives of people of color whose experiences represented the intersections of racism, classism, homophobia, and transphobia and their consequences. The analysis further revealed how other experiences demonstrated consistencies and differences based on variations in racial, class, and other identities.

### Carole: “I Do Not Have Permission to Exist as I Am.”

Carole is a Black queer woman with Caribbean origins who has lived much of her adult life in Canada. She is an artist, entrepreneur, and university student and supports herself with contract jobs and some financial help from relatives. Carole described growing up in a homophobic social environment but feeling loved and safe in her family. Her family was mindful of protecting her from harassment and violence that might occur because of her sexual identity.

Years earlier, Carole was sexually assaulted by two men who lured her to a house on the premise of presenting a job opportunity. During the rape, they referred to her sexuality and their intent to fix it. She reported the assault and said the police were sensitive and helpful, however, neither of the perpetrators faced justice. She had disclosed this experience to a counselor and a few friends, while choosing to protect her family from knowledge of it. She described long-term consequences of the assault:

*I tend to react very angrily. I shouldn’t say that – because I don’t address that to another person, but I do feel this instantaneous rage sometimes. If I feel like someone is trying to step on my toes, pull something over on me, there's this fear about that need to be in control over myself at all points in time. And I need not to have people around me who are going to try and take that control away*.

She was also aware of the wider implications of this assault:

*I hear stories on the news right now – in South Africa, corrective rape that's going on. Corrective rape in Iran and in different parts of the world. It's going on here. I just, um, I feel helpless when I hear about it. I don’t know what I can say about it. There are just certain people that have it in their mind that I do not have the permission to exist as I am. I’m supposed to toe a particular line, and if I’m not … And this is for women in general, not just lesbians. If you are not behaving yourself and doing what's expected of you, there are repercussions for it, right*?

In her interview, Carole recounted the recent experience of being approached by a man who had been at the house where she was assaulted; he had left her with the other two men when he was told of their plan. She had not seen this man since the night of the rape. He had approached her seeking absolution for his decision to abandon her, impressing on her that he had been distressed about the decision ever since. Carole shared how the aftermath of this encounter was another episode of depression, the latest in a series of illness episodes that had sometimes brought her to the point of contemplating suicide. She described working with a counselor for several years but sometimes needing to wait months for service because she cannot pay for counseling not funded by the government health insurance plan. She explained how she prioritizes seeking help because, “*People who don’t get help kill themselves, you know, it's that simple. They either do it outright through suicide, or they do it through drinking themselves to death, or taking drugs.*”

Carole's assault is a very direct example of violence being used as a tool of oppression: the perpetrators positioned the violence as a method for disciplining what they saw as Carole's gender-transgressive queer sexuality and identity. Their sense of entitlement to do so was communicated by their openness in sharing the plan with the other man who was in the house. Such behavior suggested their expectation of impunity for their actions. Lake (2021) points out that, internationally, sexual assault against lesbian women is sanctioned tacitly by public discourses surrounding “corrective rape”. The method may be stigmatized but the rationale for “correction” is unquestioned. The associations between specific sexual or gender identities and exposure to violence is not interrogated. Attention is to perceived deviances of victims rather than demonstrated deviance of perpetrators.

These problematic discourses are a context for the assault, however, Carole was also put in harm's way by economic circumstances in which her work was precarious, compelling her to be in a situation of potential risk to pursue a livelihood. Blosnich and Goldbach (2020) suggest that sexual minority women may be disproportionately targeted for sexual violence because predators perceive them as alienated from peers and, therefore, unprotected. An intersectionality framing would extend that hypothesis to suggest that sexual minority women who are also racial minority women or also economically marginalized women, may be perceived as alienated and disenfranchised from society as a whole and, therefore, less able to mobilize protections or responses that would reduce their vulnerability to predators or would hold predators accountable. Therefore, it is possible that the assailants judged Carole more assailable in a context where violence against racialized bodies is not afforded the level of response that is attained by violence against White bodies (Williams, 2016).

For Carole, the recent encounter with a complicit witness, combined with a forced revisiting of the trauma, was another violence. The man who accosted her sought compassion for his pain about the role he played in the assault; he asked that she reciprocate the compassion he claimed to have for her. The violence of confronting her with this revisiting of the assault is furthered by the epistemic violence (Dobson, 2011) of pressuring her to rewrite her narrative to specifications that reduced his accountability. His potential ignorance of the ways in which he further assaulted and traumatized Carole did not make it any less harmful. The retraumatization of the encounter and the disempowerment Carole may have felt in it provides a context for the decline into depression that followed.

The perception that violence was punishment for embodying certain identities was common in the narratives of participants. It was described in ways that highlighted the interviewees’ internalization of cultural scripts that made them culpable for not conforming. It also demonstrated participants’ awareness of the pervasive presence of violence for sexual minority women and trans people. For example, a trans woman listed off experiences that made it hard to face dealing with the world: “*Being called names, being teased … People have thrown cigarette butts at me. People have spit at me. I’ve even had this one lady try to run me down with her car.*” An Indigenous lesbian woman spoke of “*People beating me up. All kinds of shit. Bashing happens. You’re a woman and violence affects your life. Sexual assaults happened.”*

However, intersections of oppression and privilege were also visible in the narratives. There was an awareness that the capacity to pass as a member of dominant groups afforded protection from violence. For example, a trans woman who usually presents as a man explained, “*No [violence], not at all. Because like I said, I haven’t been out.*” The Indigenous woman quoted above also expressed gratitude for not experiencing more violence, noting “*I feel guilty for feeling thankful for it … I very much have White privilege. I think most people would see me as poor, rather than mixed or Native*.”

In her account, Carole suggested that violence can be visited upon certain people for existing, and the violence can continue beyond a specific assault or threat. Putting Carole's account in the context of the other narratives, it is apparent that participants were attuned to cultural contexts that granted permission for existing to some and denied that permission to others. In addition, those who were able to avoid violence by emulating the dominant (e.g. White or male) protected themselves by doing so; individuals took on the work of strategizing for their safety. The belief that violence is based on how individuals embody their sexual or gender minority identities reflects the dominance of cultural scripts affirming that violence is an inevitable outcome for people living within these identities (Lake, 2021). However, violence is made possible because those cultural scripts grounded in homophobia and transphobia are reflected in institutions and systems that undermine attempts to make a safe life, and fail to protect people from perpetrators of harm.

### Rory: “It's Not the First Time I’ve Been Sexually Assaulted. The Ninth Time.”

Rory is a Black trans man who has been connected to social services since childhood. At the time of interview, he was aging out of youth services. His association with the social service system began with child welfare involvement due to physical abuse in his family home and continued into time in youth shelters when he ran away from that home. In his family home, at school, and in the shelter system, he described being bullied for not conforming to gendered expectations. At the time of interview, he had connections to the health and social service systems associated with a disability that he was cautious about disclosing to others. He was in the process of completing the institutional tasks that are associated with gender affirmation, however, was unable to afford any medical interventions. Rory had a long history of mental health crises including suicide attempts and hospitalizations. He described struggles to find connection with others and past relationship breakdowns that had prompted acute episodes of depression. In addition to describing depression in the past year, he reported excessive alcohol use. His reason for drinking was summarized as “*To hide how I am feeling. To make the pain go away*.”

Rory described long-term consequences from the violence he experienced in his family home, and later as a street-involved youth. The recurrent exposure to violence at a young age and since had resulted in a need to ward off panic whenever people come close to him. He said he had developed a menacing stance to keep people at a distance, but that it had costs: “*I hate being afraid. I hate being lonely*.” A recurrent motif in his interview was the belief “*Nobody gets me … nobody is gonna take the time to get to know me.”*

A sexual assault that Rory had experienced in the past year, perpetrated by a neighbor who was known to him, was the latest in a series of sexual assaults starting from childhood. Referring to the last assault he said, “*It's not the first time I’ve been sexually assaulted. The ninth time.*” He explained that the assault was reported to the police, but no one had helped him deal with the assault or the depression that followed. Although Rory was a client in multiple health and social services, his reported experience was that no help was available to support him through this experience.

Rory's account described persistent violence and threat associated with responses to his sexuality and gender identity. Racism and ableism may be implicated in some of these experiences as well, but his narrative did not make those connections. Instead, his account raised questions about how and why various systems have failed to protect him from violence. Violence in his childhood home began a series of institutional involvements that were purported to provide safety. Instead, institutional interventions left him in a home that he had to flee, then in shelters, then in housing where, again, he was not safe. Structural violence was revealed in the failure of several systems to protect this young, Black, trans man living with a disability from danger and, most recently, the failure of more systems to provide help when violence happened. The healthcare system responded to his most extreme needs when they were represented in the possibility of suicide, but his experiences of despair, isolation, and vulnerability were unattended, by his account. Although a series of institutional failures formed the context for his suffering, Rory described how those institutions took little responsibility for addressing the consequences.

There were several participants who likewise described histories of violence and victimization beginning from childhood and recurring into adulthood with long-term mental health consequences. A bisexual woman described the impact of abuse and assaults experienced from her teenage years through adulthood, noting “*I don’t even think I would have depression at all if I hadn’t had that happen.*” Another woman who preferred not to classify her sexuality made a more indirect connection, suggesting “*…The violence aspect – I don’t think my anxiety and depression now is because of that, but it's certainly part of the life experiences that shaped who I am*.” However, participants noted a significant impact from living with the knowledge that violence was recurrent and had lingering consequences for mental health and well-being. A White bisexual woman who was able to speak at length of the privileges she carried because of race, education, and socioeconomic status, still noted that her experience of childhood sexual abuse and then other violence in relationships had a long-term effect:

*I think that I was a bit of – sort of sleepwalking through the first however many years of my life, to a certain degree, emotionally. And so, sort of like – walking wounded … it was prolonged, repeated, and had, I think, a very significant impact on my development – emotional and mental development. It's, I think, it informed a huge part of who I am, both good and bad, and presented certain challenges*.

These narratives of women and trans people who have experienced violence multiple times by multiple perpetrators over a lifetime are consistent with research suggesting that violence against women and gender minority people can be recurrent (Bauer & Scheim, 2015; Logie et al., 2014). These multiple victimizations bring women and trans people into contact with healthcare institutions, social and community services, and justice systems. Applying a structural analysis turns attention to how each assault was an opportunity when victims could have been protected and were not. Each assault was an opportunity when victims could have been transferred to circumstances of safety and were not. Instead, these individuals move through life carrying the burden of recovery from these assaults. They carry the relational, emotional, and mental consequences of the associated traumas, and hold the tension of waiting for more violence to come because they are not protected by the institutions that should be keeping them safe.

### Brenda: “I Don’t Go Out and Seek These Experiences. They Seem to Find Me.”

Brenda self-identifies as an Indigenous Two-Spirited woman who is a mother, a university student, and a volunteer in advocacy work for LGBTQ and other communities. She described herself as having “masculine tendencies” that she hides by presenting herself as “extremely feminine” to avoid harassment. In addition, Brenda grew up in a home where there was violence and she, her mother, and her siblings were all abused. Later, as an adult, she had abusive partners, and she described being fearful of this possibility whenever she starts a new relationship.

Brenda described a life that is defined by entering and leaving circumstances to find safety for herself and her daughter. At the time of interview, they had recently spent time in a shelter after leaving a home where another person in the house posed a threat. She had adapted to a routine of repeated resettling, finding work, and finding community. Her drive to find safety and stability for her family was also a mission to avoid the scrutiny of child welfare agencies, because she had experienced that involvement and did not want it for her daughter.

Brenda supported her family with income from part-time work and help from a family member. She described how she would like to increase her level of paid employment but balances how much she can work against how much it will cost to provide childcare for her daughter. Past employment included work as an escort and, in that job, receiving threats and assaults was routine. Yet, she never reported this violence because,

*My only thought was, can I make the rent? Do I have enough groceries? Can I put gas in my car? Can I put clothes on my back and my daughter's? My tuition is due next month …. You know what I mean?*

Brenda was self-aware about her resilience; she credited her survival to being a strong woman who has some people who care about her. Reflecting on the recurrence of violence and harassment in her life she said, “*I don’t go out and seek these experiences. They seem to find me. I don’t know why. They have followed me my whole life.*” However, she also stated, “*That's what you get when you’re a woman that dresses what men would consider suggestive in this society.”*

Brenda explained how she has been reluctant to share these experiences with others or to seek help. Just as she has learned to hide her Two-Spirit identity to avoid harassment, she hides her precarious circumstances from others, as described here:

*They thought I was a very intelligent, um, upwardly-mobile, confident young woman who was trying her best to be a good mom. That's all they saw me as. And I let … I sort of let them … and plus, I didn’t feel like they … I didn’t have a lot of confidence that they had a lot to offer me anyways*.

Brenda believed she was affected by depression and post-traumatic stress disorder, noting “*I don’t really have a clear understanding of PTSD. It's just that by gauging all of the violent experiences I’ve had in my life, I’d pretty much figured that*.” She had not been diagnosed because her attempt to seek assessment was cut short when the male psychologist sexually harassed her. She recounted a story in which he asked her to come to his home for an appointment, offered alcohol and made sexual advances, and then charged her for the appointment that she had terminated immediately. It frustrated her that she saw no way to hold this man accountable for his behavior. She described being reluctant to seek help again and, instead, said she self-medicates with exercise, diet, and journaling and, sometimes, alcohol.

Brenda described a life of chronic exposure to danger, abuse, and assault. She went from being a child at the mercy of a dangerous man, to being a woman vulnerable to danger from a series of men who were intimate partners, clients, and a helping professional. An insidious effect of this history was that she defined herself as a person stalked by violence and concluded that depression and trauma were burdens she must carry. As with Rory and Carole, her embodied experience as a woman, Two-Spirit, and Indigenous were marked by being put in harm's way and made assailable without apparent consequences for those who perpetuated violence against her. In addition, she was compelled to accept work that required routine exposure to danger in exchange for sufficient income and resources to care for her family. The violence she experienced in intimate relationships and sex work was recurrent and, by her account, she did not experience systems that would help or safeguard her. In both intimate partner violence and sex work, women often face social and institutional responses that shame and stigmatize them while withholding protection or justice (Thaller & Cimino, 2017). In Brenda's story, multiple systems were implicated in her vulnerability to harm and the long history of depression.

Brenda's words revealed a cultural context in which she was made to carry shame, to feel responsible for her victimization, and to hide her identity and her circumstances to avoid the scrutiny that could lead to stigma and harmful institutional encounters. Other participants shared similar concerns about how the violence they faced could lead to detrimental institutional encounters. For example, a trans person shared that although aware of services available to persons facing violence, stigma stopped them from seeking help: “*I went for over a year in my last abusive relationship because I didn’t want to go to a shelter because I didn’t want to be the person in the shelter*.” A middle-aged bisexual woman noted that she did not seek help for the mental health consequences of childhood sexual abuse because “*If it ever came to a court case between me and my father, those notes could be used against me*.” Structural violence is revealed in institutional systems that sexual minority women and trans people fear will fail to support them or will punish them for being victims of violence. With institutional involvement either dangerous or absent, individuals are left in circumstances of violence, and live with consequences for their mental health.

### Mental Health Care System Contributions to Structural Dimensions of Gender-Based Violence

When participants spoke about their experiences seeking mental health services, they recurrently presented a system that was another source of trauma for sexual minority women and trans people. This issue was so pervasive in the interviews that it is represented in this section by the collective instead of a single case study.

As referenced in the narratives above, many could not access care because government-funded services were scarce, and private sector services were unaffordable. In addition, violence, gender-based discrimination, and mental health problems were reasons that people could not access or maintain secure employment, housing, education, and childcare, making it more difficult to access services. Through their accounts, participants revealed a healthcare system that expected them to be personally and financially responsible for the outcomes of interpersonal and structural violence.

The interviews’ emphasis on experiences of mental health care resulted in much of the commentary about services addressing micro-level components of service provision, for example, availability of sexual minority and gender minority service providers and preparation of service providers for addressing LGBTQ issues. However, there were two areas in which the participant accounts described institutional practices that reinforced structural inequities contributing to systemic violence against sexual minority women and trans people: the stigma associated with being victims of violence, and the unavailability of mental health supports for LGBTQ people who are recovering from trauma.

Participants described treatment experiences that stigmatized them as victims of violence. For example, problematic ideologies and attitudes were reflected by practitioners who probed details of violent incidents, as if they expected the individual to provide an explanation for their victimization in the details of their personal choices. Such practices could be both traumatizing and stigmatizing. As a bisexual woman recounted, she became increasingly frustrated by a therapist who would bypass discussing her experience, instead devoting sessions to discussing the potential problems of the abuser: “*She’d get into almost analyzing him and saying, wow he must be so troubled.”* As this dominated their sessions, she was left feeling shamed for attending to her pain without attending to the potential suffering of her abuser. Stigmatization was also revealed in a trans man's story of feeling his disclosure of ongoing trauma from childhood sexual abuse was minimized by a therapist who, “… *just batted that off as – ‘Every other person that I interview has a sexual abuse history. ‘That's normal,’ he said. And it just felt like my experience had just, in a sentence, been discounted as relevant*.”

Some participants also described feeling silenced by practitioners who avoided the discussion of violence, as if unable or unwilling to acknowledge it. A nonbinary trans person recounted experiences with therapists who were, “*… skirting the issue … If you’re going to a mental health professional for help because of trauma, then they are obligated, it seems to me, to at least attempt to go near the subject*.” Brenda described her wish for people in services to not respond in ways that made her feel damaged and repulsive: “*I’m not expecting somebody to be, like, you know, super-human or anything strange like that. I just want somebody to … not cringe, like, ‘cause I told you some very traumatic things that happened in my life*.” These experiences point to ways that encounters with service providers were aligned with other institutional experiences of being silenced.

Many participants asserted that help was not forthcoming when they experienced violence or when they later sought support. They suggested that systems of care could be unresponsive to people seeking help to deal with the long-term impact of violence, even though many people need to wait years before feeling ready to seek help. For example, Carole noted that she was not ready to seek support right away, because “*I just wasn’t interested in any of that. I was at the point where, it happened, I don’t ever want to talk about it or deal with it again. That was how I dealt with it.*” People who had experienced childhood sexual abuse also asserted that it took years for them to seek help and then required years for them to work through the consequences of the abuse. The woman who was quoted earlier describing herself as “walking wounded” said “*That healing journey took a long time – probably about 10 years or more, to come to terms with that*.”

Once they were ready to seek help, individuals reported rationing their access to mental health services, knowing it was a scarce resource that might not be available when needed. There was also concern about access for others, as described by Carole, who limited her sessions to once a month, “*I know other people need these spaces as well, so I’m just trying to take my fair share.*” This scant access to mental health support stands in contrast to knowledge that the recommended best practice for treatment of complex trauma is multi-phased, regular access to psychological care (Cloitre et al., 2011). Participants’ accounts of the under-resourcing of services for LGBTQ people seeking psychosocial support for violence and trauma suggests a structural inequity that could be contributing to their prolonged distress and poor mental health.

## Discussion

This study analyzed the narratives of sexual minority women and trans people to examine the structural and cultural mechanisms underlying their accounts of violence and depression. The stories the participants shared revealed that violence was not limited to a particular point in time or relationship, but rather was a recurrent experience through a lifetime, occurring across different interpersonal contexts and institutional encounters. They lived in a state where violence was always possible. In their accounts, justice was unattainable. Analysis of their experiences reveals that their victimization and impunity for perpetrators was made possible by structural processes aligned with interlocking racism, misogyny, homophobia, and transphobia.

When these participants sought help or wanted accountability for the people who had harmed them, justice systems and healthcare systems minimized or avoided dealing with their needs. In addition, these participants shared the emotional impact of cultural scripts and institutional practices that compelled them to accept responsibility for the violence they endured. The violence and the institutional failures surrounding it were a core element in what people experienced as mental health struggles, and their difficulty accessing appropriate health care and supports as sexual and gender minority people intensified those struggles.

The stories and experiences presented in this study trace a path from wounded bodies representing as depression to wounding cultural norms and wounding structures. Violence against sexual minority women and trans people is made possible by a cultural context that socializes men and others to feel entitled and sanctioned to exert violence as a consequence for gender identities and performances judged transgressive (Jewkes et al., 2015; Kelley & Gruenewald, 2015). The assault experienced by Carole is only one example of how physical, verbal, and emotional violence is used to discipline those who do not conform (Blosnich & Goldbach, 2020; Doan-Minh, 2019). Carole's story represents how cultural norms and social practices empower perpetrators of violence and disempower their victims.

In addition, this cultural context fosters discourses in which victims are socialized to take responsibility for their victimization and to do the work of absolving perpetrators and others of responsibility. It is this culture that allows a man who aided Carole's rape to demand compassion, makes Rory accept that no help or understanding is coming to him, and prompts Brenda to reconcile herself to the male gaze as a precursor to violence. These cultural discourses are reinforced by structural arrangements and institutional practices that systematically place the bodies of sexual and gender minority people in harm's way.

Carole, Rory, and Brenda's stories are stories of precarious employment and income insecurity. Rory and Brenda's stories are stories of inadequate access to healthcare and inadequate support from social service systems. All three stories reveal justice or other systems that do not hold perpetrators accountable. Cultural scripts that stigmatize individuals align with social and economic circumstances, institutional practices, and policies that place people in situations that are unsafe, and treat the occurrence/recurrence of violence against them as expected, deserved, and not worthy of response (Montesanti & Thurston, 2015). These are the cultural and structural mechanisms that allow systemic violence against sexual minority women and trans people, resulting in lives of trauma and depression. When systems further fail people by under-resourcing systems of care and constraining access to mental health services so they are beyond the reach of those that are economically marginalized, it adds another layer to the links between structural violence, interpersonal violence, and depression (Rao et al., 2012).

Although the sample for this study was mostly White-identified, the narratives chosen for focus in this article were from people who identified as Black and Indigenous. The selection of these individuals as representatives is an opportunity to contemplate the specific consequences of anti-Black racism and anti-Indigenous racism in their intersections with (cishetero)sexism, homophobia, and transphobia. These narratives demonstrate that intersectionality is not just an analysis of the convergence of identities but is also an analysis of convergences of structural and institutional oppression (Collins & Bilge, 2020; Crenshaw, 1991). The accounts are illustrative because they suggest the possibility of “structural intersectionality” (Few-Demo, 2014) that subjects people who are located in intersecting vectors of oppression to multiplicative experiences of domination, exclusion, and harm in social and institutional spaces. Blackness and Indigeneity are consequential in justice systems, child welfare systems, healthcare systems, and labor markets. Those consequences are further extended by simultaneous exposure to institutional dynamics that exclude sexual and gender minority people.

Multiple systems converged to put Carole, Rory, and Brenda in harm's way and converge to put other sexual minority women, trans people, racial minority, and Indigenous people in harm's way. Awareness of the intersectional consequences of structural violence are increasingly visible in the public sphere, recognized as determinants of life trajectories for marginalized people. 11-year-old Naomi Wadler makes speeches where she names murdered Black girls and women and tell us how systems converge to allow harm to Black women and girls without public notice (https://youtu.be/C5ZUDImTIQ8). 12-year-old Nevaeh Pine has posted a video where she names murdered Indigenous women and girls and tell us how systems converge to allow the disappearance and murders of Indigenous women without public attention or response (https://youtu.be/qffVAhRiFBk). These young girls assert that minoritized race and gender are not the cause of victimhood but, instead, are the determinant of whether systems of protection are mobilized to one's benefit. Lake (2021) extends this analysis to Black lesbian women, noting Butler's indictment of power relations that render such people “injurable” and “lose-able.” In turn, this study asserts that the risks faced by its participants were defined by power relations that made them injurable by constructing barriers to the housing, income, employment, and social status that would protect them from violence.

Structural dimensions of violence against sexual minority women and trans people were also revealed in the experiences that people had when they sought support. Participants described help-seeking experiences that were shaming and silencing. The encounters they described represented their own violence, specifically, they were manifestations of less tangible but still harmful epistemic violences that stifle testimonies, silence voices that seek justice, and insist on the rewriting of narratives to diminish their consequence (Dobson, 2011). Challenging the structural dimensions of violence against sexual minority women and trans people calls social workers to action that shifts the culture and practices of health and social service organizations toward increased access for these populations, and equipping practitioners to deliver services that are trauma-sensitive and trauma-centered (Knight, 2015; O’Dwyer et al., 2019). Practitioners need to be attentive to violence as a context for people's distress and mindful of how structural dynamics manifested in institutions, including health and social services, can be a further context for mental health struggles. Yet, these case studies also point social workers toward the need for advocacy across sectors for safe, affordable housing, for safe employment that pays adequate wages, for justice responses that prioritize restoration for victims as well as accountability for perpetrators, and for destigmatizing histories of violence. If we understand the heightened possibility of violent victimization for lesbian, bisexual, and trans people is based on social, economic, and political arrangements that harm and allow harm, then social workers need to work within and across institutions and sectors to shift these structural processes that put sexual minority and gender minority people in positions of danger. The work of advocacy, system reform, and civic and global engagement are necessary to address violence and seize the opportunities to prevent violence. Further, social workers must be prepared to work within the profession, within communities, and within institutions to challenge the cultural scripts that individualize gender-based violence with consequences of stigma, social isolation, and depression for those affected.

## Conclusion

This article provided a glimpse into the lives of sexual minority women and trans people who experienced recurrent violence. That violence was revealed to be associated with long-term experiences of depression and injustice. In their accounts, structural and cultural arrangements positioned them in harm's way and intersections of oppression and privilege determined access to safety, justice, and support for their mental health. A key learning from the analysis is that it is incomplete to treat gender-based violence as an individual problem without also attending to the systemic violences that contribute to its prevalence.

Multisystemic interventions must be informed by research to understand the structural determinants of violence and their manifestation in the lives of people who live within intersecting vectors of oppression. This study was limited because a secondary analysis was imposed on a dataset that was designed primarily to explore access to healthcare for sexual minority women and trans people. Future research needs to center on intersectional experiences and explore the structural dimensions of violence more deeply. Work is needed that delves further into the politics of violence, traces structural violence enacted through institutions, and surfaces the intersecting and specific experiences of those that are affected by racism, poverty, disability, and other marginalized experiences. That is the work that will inform anti-violence social work, anti-violence organizational practices, and anti-violence policy reform that is responsive to people in our community who are targeted to greater degrees by the interlocking effects of structural violence.

Although this is a small sample study that cannot speak to the general experience of sexual minority women and trans people, the stories these participants shared were an indictment against a society that structures inequitable access to resources and services and creates an environment for violence, denied justice, and withheld help. This article is a call to social workers and community members to insist that violence against sexual minority women, trans people, and other groups be viewed intersectionally, and in structural context. That analysis is the start of transforming cultures and systems that allow it.
